# Therapeutic Effectiveness and Safety of Mesotherapy in Patients with Osteoarthritis of the Knee

**DOI:** 10.1155/2018/6513049

**Published:** 2018-01-04

**Authors:** Liang Chen, Dongqing Li, Jun Zhong, Bo Qiu, Xianglei Wu

**Affiliations:** ^1^Department of Orthopaedics, Renmin Hospital of Wuhan University, 9 Zhangzhidong Street, Wuhan, Hubei 430060, China; ^2^Department of Microbiology, School of Basic Medical Science, Wuhan University, 185 Donghu Road, Wuhan, Hubei 430071, China; ^3^Institute of ENT, EENT Hospital of Fudan University, 83 Fenyang Road, Shanghai 300021, China; ^4^Laboratory of Immunology, University of Lorraine, avenue du Morvan, 54511 Vandoeuvre-lès-Nancy, France

## Abstract

**Objective:**

To evaluate the therapeutic effectiveness and safety of mesotherapy by comparing it with the classic systematic therapy in patients with osteoarthritis (OA).

**Methods:**

Sixty patients were included and classified into two groups based on the existence of contraindications for nonsteroidal anti-inflammatory drugs (NSAIDs). These patients were treated with oral NSAIDs (Group A) or mesotherapy (Group B). After completing the treatment, the patients were followed up for 6 months. Their clinical features, laboratory results, and Western Ontario and McMaster Universities Osteoarthritis Index (WOMAC) scores were evaluated.

**Results:**

A total of 50 patients completed treatment and follow-up. The patients in Group B had significantly fewer gastric acid-related complaints and requested less supplementary treatment for recurrent pain (*p* < 0.05). The patients in both groups exhibited decreased blood viscosity after treatment (*p* < 0.05). WOMAC scores, specifically those for pain and stiffness, were found to be significantly improved after either type of treatment (*p* < 0.05). Mesotherapy also ameliorated physical function (*p* < 0.05). Furthermore, Group B presented with better outcomes than Group A (*p* < 0.05 or *p* < 0.01).

**Conclusion:**

Our results suggest that mesotherapy is an effective and safe treatment for patients with OA. Clinicians should consider mesotherapy as an alternative therapy for patients with contraindications for NSAID use.

## 1. Introduction

Osteoarthritis (OA) is one of the most common degenerative diseases of the musculoskeletal system. In China, the prevalence of symptomatic knee OA is 15.5% among women and 5.6% among men; similar prevalences have been obtained among Caucasians in the USA [1]. The burden of OA is related to both its prevalence and its cost to the healthcare system. March and Bachmeier [2] found that the annual cost for musculoskeletal disorders has been estimated to range from 1 to 2.5% of the gross national product (including in the USA, UK, France, Canada, and Australia).

OA presents as a combination of forms of joint damage involving the degeneration of cartilage, deficits in repair of the cartilaginous tissues, and remodelling of the bone via chondral and synovial secondary reactions [3]. OA is always extremely painful for the patient, causing loss of ability and often stiffness. The goal of OA therapy is to relieve these symptoms and improve quality of life while minimizing medication-related adverse events [4]. Traditionally, acetaminophen/paracetamol is the most frequently used analgesic recommended as the first-line treatment for mild OA of the knee. For moderate to severe symptoms, this drug appears to be less effective than nonsteroidal anti-inflammatory drugs (NSAIDs) [5]. Recently, a meta-analysis suggested that diclofenac 150 mg/g instead of single-agent paracetamol is currently the most effective drug for OA in terms of improving both pain and function [6]. However, the side effects of NSAIDs, such as gastrointestinal bleeding, combinational risk, and increased cardiovascular risk, have constrained the application of these drugs for certain patients with coexisting disorders.

Mesotherapy refers to the intra- or subcutaneous injection of active compounds to treat local medical and cosmetic conditions. Via these intradermal microinjections, mesotherapy allows drugs to exhibit direct and prolonged local pharmacological activity in the skin [7, 8]. Because intradermal administration and the use of lower drug doses decrease the risk of systemic interaction, mesotherapy has been shown to have significantly reduced side effects relative to traditional treatments. In 2011, a panel of specialists reached a consensus regarding the scientific rationale, advantages, indications, and contraindications associated with the use of mesotherapy [9]. Mesotherapy provides an alternative therapeutic strategy for the management of locoregional disorders, particularly with respect to controlling pain [10]. Prior research involving the use of anaesthetics, NSAIDs, muscle relaxants, and other analgesics has indicated that mesotherapy could reduce pain (cervical and back pain and tendinopathy) by at least 50% relative to baseline [11].

However, additional clarification is required regarding the question of whether mesotherapy for OA of the knee has additional benefits beyond reduced side effects. In the present study, we investigated the therapeutic effects of mesotherapy by comparing this treatment with oral NSAIDs. We sought to enrich evidence for the clinical application of mesotherapy via this controlled study.

## 2. Materials and Methods 

### 2.1. Patients

Between May 2016 and June 2017, a total of sixty patients who had been diagnosed with OA were recruited and treated at the Department of Orthopaedics of Renmin Hospital of Wuhan University.

The following criteria (derived from [12]) were used to diagnose OA of the knee:

(1) knee pain with at least 5 of the following characteristics: age > 50 years, stiffness for <30 minutes, crepitus, tenderness in bony regions, bony enlargement, a lack of palpable warmth, ESR < 40 mm/hour, RF < 1 : 40, and synovial fluid signs of OA (in clinical and laboratory assessments);

(2) knee pain with osteophytes and either age > 50 years, stiffness for <30 minutes, or crepitus (based on clinical and radiographic features).

Thirty patients without known contraindications for NSAIDs were organized into Group A. An additional thirty patients were organized into Group B; this group received mesotherapy. Patients with a known hypersensitivity to certain products and patients who had received another therapy (such as an infiltration of corticosteroids or physical therapy) within 5 weeks before the start of the study or a surgical intervention within 3 months prior to the study were excluded. Clinical characteristics and laboratory results were collected for all the included patients before and after treatment. The follow-up for each patient lasted for 6 months. All patients provided informed consent.

### 2.2. NSAIDs

In Group A, the initial treatment prescribed to patients was the oral administration of diclofenac 75 mg twice per day for the first 3 months and then upon request.

The patients in this group were permitted to take either misoprostol or proton pump inhibitors if they felt dyspepsia, heartburn, nausea, or bloating. Patients were removed from the study when any sign of gastrointestinal bleeding (e.g., melena) was confirmed or when another treatment was received.

### 2.3. Mesotherapy

**Figure 1:**
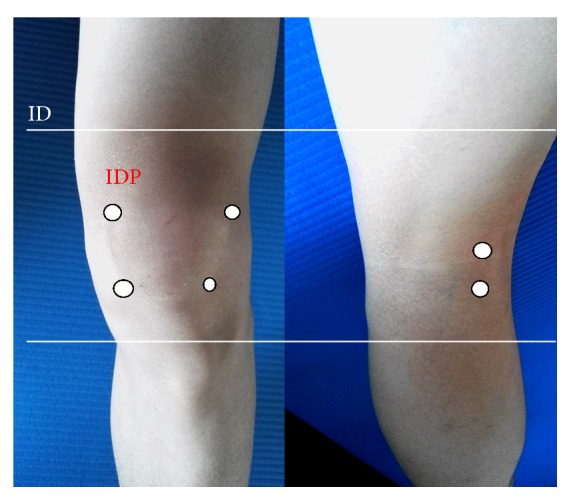
Injection sites used for mesotherapy. IDP was administered at 6 points along the joint space (4 anterior and 2 posterior). IDS was administered to 4 faces of the knee.

The patients in Group B were not allowed to take any oral analgesics or corticosteroids. The materials used for mesotherapy included disposable sterile syringes (BD®), sterile single-use needles (0.26 mm × 4 mm and 0.3 mm × 13 mm, Terumo®), a disinfectant, and consumptive products. Two mesotherapy protocols were utilized depending on the disease status. For patients in the acute phase, the following substances were mixed: 2 ml of 1% lidocaine, 40 mg of piroxicam in 2 ml, and 100 U of calcitonin in 1 ml. There were sessions on D1, D8, and D15 and upon request thereafter. For patients in the chronic phase, a mixture of 2 ml of 2% procaine, 2 ml of organic silica (Conjonctyl®), and 100 U of calcitonin was used. There were sessions on D1, D15, D30, and D60 and upon request thereafter. A mix of injection techniques, including IDP (profound intradermic injection; injection depth = 2–4 mm) and IDS (superficial intradermic injection; injection depth = 1-2 mm), was administered during each session for both protocols. Illustrations of injection sites are presented in Figure 1. All the treatments were formulated by two qualified mesotherapists and administered by the same physician.

### 2.4. Evaluations

The therapeutic efficacy and safety of the two treatments were evaluated for each patient at the end of the 6-month follow-up (M6) with comparisons with baseline findings (M0). The major therapeutic outcome was measured using the Western Ontario and McMaster Universities Osteoarthritis Index (WOMAC), which includes five items for pain (score range: 0–20), two items for stiffness (score range: 0–8), and 17 items for functional limitation (score range: 0–68) [13]. Side effect-related symptoms (including allergy, dyspepsia, heartburn, nausea, bloating, and melena) were recorded to assess the safety of the administered therapies. Laboratory tests were also conducted at M0 and M6 to evaluate the two aforementioned aspects of treatment.

### 2.5. Statistical Analysis

All data were analysed using SPSS 22.0 software (Chicago, USA). Count data were expressed as numbers of cases and percentages. Measurement data are expressed as the mean ± standard deviation ($\overset{-}{x}$ ± SD). Independent samples *t*-tests and chi-square tests were used for statistical analyses, and *p* < 0.05 was set as the threshold for significance.

## 3. Results

### 3.1. Patients' Clinical Characteristics

Six patients who were originally in Group A were excluded from the study (2 patients were lost to follow-up, 2 patients received a corticosteroid infiltration, 1 patient exhibited melena, and 1 patient had itchy skin). In Group B, a total of 26 patients were included (4 patients were lost to follow-up). Patients' baseline and posttreatment clinical features are summarized in Table 1. Compared with the patients in Group A, the patients in Group B had significantly fewer gastric acid-related complaints (*p* < 0.05) and requested less supplementary treatment for recurrent pain (*p* < 0.05).

### 3.2. Haemorheology

Table 2 lists dynamic changes in haemorheological parameters. Both NSAIDs and mesotherapy significantly reduced blood viscosity (*p* < 0.05) but did not significantly alter other haemorheological parameters. A lower erythrocyte aggregation index after treatment was also observed in patients in the NSAID group but not in those in the mesotherapy group (*p* < 0.05). No significant differences between Groups A and B were observed with respect to haemorheological parameters.

### 3.3. Outcome Assessment

**Figure 2:**
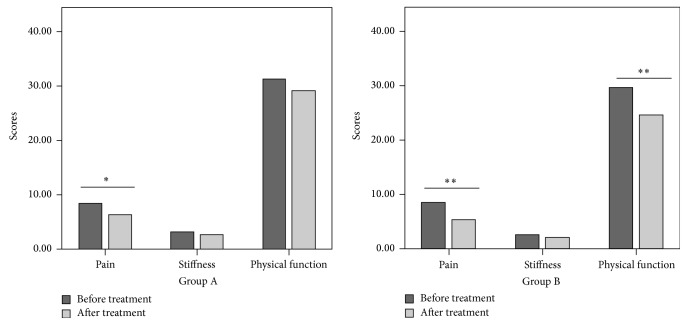
WOMAC evaluations of the patients' clinical outcomes. ^*∗*^*p* < 0.05; ^*∗∗*^*p* < 0.01. WOMAC: Western Ontario and McMaster Universities Osteoarthritis Index. This index includes five items for pain (score range: 0–20), two items for stiffness (score range: 0–8), and 17 items for functional limitation (score range: 0–68).

As indicated in Figure 2, for both groups, improved pain scores were observed for patients after treatment (*p* < 0.05 or *p* < 0.01). The patients in the mesotherapy group also showed ameliorated physical function (*p* < 0.05). There were significant therapeutic effects of treatment for the patients in the mesotherapy group relative to the patients in the NSAID group (*p* < 0.05 or *p* < 0.01).

### 3.4. Subgroups Analysis

To eliminate the interference of the inflammatory phase, four subgroups were created and compared. With regard to dynamic haemorheological changes, inflammation status was an independent factor (Table 2, acute phase: Group A versus Group B, *p* < 0.05, blood viscosity and erythrocyte aggregation index; chronic phase: Group A versus Group B, *p* > 0.05).

The initial status of inflammation did not show impact on WOMAC evaluations. Patients in both acute and chronic phases presented significant therapeutic effects of treatment (acute phase in Group A versus acute phase in Group B, *p* < 0.05; chronic phase in Group A versus chronic phase in Group B, *p* < 0.05).

## 4. Discussion

Here, we have evaluated the effectiveness and safety of mesotherapy in patients with OA of the knee by comparing this therapy with traditional NSAID treatment. Based on our results, this study showed that both NSAID treatment and mesotherapy significantly improved patients' biochemical markers and clinical conditions. Notably, relative to NSAID treatment, mesotherapy had significantly fewer side effects and was more efficient in terms of haemorheology and WOMAC scores.

Cartilage degeneration is an important pathological feature of OA. Synovial inflammation is the central component of this degeneration process, which causes joint swelling and pain in patients with OA. Studies have shown that synovitis and OA progression are closely related; therefore, inhibition of the development of the inflammatory response to OA treatment is essential [14]. Normally, ESR and the plasma level of CRP reflect inflammation severity and disease activity. Therefore, these metrics can be used to evaluate the clinical efficacy of antiarthritis therapy [15]. Increased blood viscosity was observed during the course of OA, and blood viscosity was reduced after either NSAID treatment or mesotherapy. Theoretically, blood flow could be increased in the affected region, and this possible outcome proves the effectiveness of the given treatment [16].

For a long period, the first-line role of NSAIDs in arthritis treatment was ensured by these drugs' anti-inflammatory and analgesic effects. In this study, we observed that both of the tested treatments had similar efficacy and that better outcomes were observed for mesotherapy than for NSAID treatment in certain respects. This tendency has also been observed in other studies that involved patients with pes anserine bursitis or acute low back pain [17, 18]. A possible explanation for the differences observed in comparisons of the two tested therapies might involve the methods by which drugs are administered. Although there remains a lack of available detection capabilities for mesotherapy, this treatment is normally hypothesized to increase subcutaneous drug concentrations and retard the pharmacokinetics of drugs [9]. Furthermore, injected agents appear to have effects beyond those of systemic NSAIDs alone. For instance, long-term analgesic activity has been observed for lidocaine delivered via local administration [19].

Moreover, pain and other symptoms always reemerge within a short period after the withdrawal of oral NSAIDs. As observed in our study, a relatively high number of requests for repeated treatment because of recurrent pain were observed among patients who had taken NSAIDs, even after 3 months of systemic therapy. However, an extremely low proportion of patients asked for an extra treatment session after a standard mesotherapy regimen.

Compared with the patients in the mesotherapy group, much more patients who received systemic therapy suffered from adverse effects of NSAIDs, and certain patients even had to withdraw from the study. Arthritis always occurs in elderly populations, and this age factor plays a large role in increasing the incidence of side effects of medications [20, 21]. However, mesotherapy provides an alternative due to its local regional advantages.

Our study had certain shortcomings. Intracutaneous concentrations of drugs delivered via mesotherapy were not determined since there are no available techniques for measuring such concentrations. Therefore, the effective dosages of NSAIDs for the two tested therapies are not comparable due to these approaches' distinct administration methods. Moreover, mesotherapy is not only a route for medication delivery but also a type of reflexotherapy. Acupuncture has been proven to be able to improve physical function and provide pain relief in patients with OA [22, 23]. The expanded recruitment of patients in a future study that will involve acupuncture treatment has been planned to elucidate these types of additional effects. Of note, few adverse effects were observed; therefore, to apply mesotherapy, informed consent is needed from the patient [24].

## 5. Conclusions

In conclusion, our results suggest that mesotherapy is both effective and safe for the treatment of patients with OA. Moreover, clinicians should consider mesotherapy as an alternative treatment for patients with contraindications for NSAID use.

## Figures and Tables

**Table 1:** Clinical features of the included patients.

	Group A		Group B		
*Gender*					
Male	3		4		
Female	21		22		
*Age*	57.2 ± 3.4		61.4 ± 6.8		
*Course of disease (years)*	6 ± 4.6		9 ± 7.1		
*Inflammation*					
* Acute phase*	17		10		
* Chronic phase*	7		16		
*Body mass index* ^*#*^	25.3 ± 3.6		24.7 ± 4.8		
*PPIs use* ^*§*^	5 (20.8%)		0		*p* < 0.05
*Supplementary treatment* ^*§§*^	14 (58.3%)		4 (15.4%)		*p* < 0.01

	Pretreatment	Posttreatment	Pretreatment	Posttreatment	

*Laboratory test*					
CRP	16.62 ± 6.01	10.25 ± 3.50	16.43 ± 5.73	9.50 ± 3.46	*p* < 0.05
Group A versus Group B	*p* < 0.05				
ESR	42.64 ± 11.77	29.31 ± 8.14	42.34 ± 10.68	21.77 ± 6.42	
Group A versus Group B	*p* > 0.05				

^#^BMI: body mass index = weight/height^2^. ^§^PPIs: proton pump inhibitors. ^§§^Supplementary treatment: a request for oral NSAIDs after 3 months of treatment in Group A or an extra session of mesotherapy in Group B.

**Table 2:** Dynamic haemorheological changes from the time before treatment to the time after treatment.

	Blood viscosity	Plasma viscosity	Hematocrit (%)	Fibrinogen (g/L)	Platelet sticky rate (%)	Erythrocyte aggregation index
*Group A*						
Pretreatment	5.6 ± 1.3	1.5 ± 0.4	43.7 ± 2.2	4.3 ± 0.9	50.0 ± 10.1	9.2 ± 4.8
Posttreatment	4.9 ± 1.4	1.4 ± 0.6	42.1 ± 2.4	4.0 ± 1.0	48.5 ± 3.6	8.1 ± 4.7
*p*	<0.05					<0.01
*Group B*						
Pretreatment	5.4 ± 1.6	1.6 ± 0.1	42.3 ± 4.1	4.7 ± 1.3	51.4 ± 13.1	9.5 ± 3.6
Posttreatment	4.2 ± 1.8	1.4 ± 0.2	40.1 ± 2.4	3.5 ± 1.4	33.6 ± 4.3	8.1 ± 4.2
*p*	<0.05					
*Group A versus Group B*						
*p*	>0.05					
Acute phase						
*p*	<0.05					<0.05
Chronic phase						
*p*	>0.05					
