# Erratum to “Therapeutic Effectiveness and Safety of Mesotherapy in Patients with Osteoarthritis of the Knee”

**DOI:** 10.1155/2018/5327589

**Published:** 2018-05-14

**Authors:** Liang Chen, Dongqing Li, Jun Zhong, Bo Qiu, Xianglei Wu

**Affiliations:** ^1^Department of Orthopaedics, Renmin Hospital of Wuhan University, 9 Zhangzhidong Street, Wuhan, Hubei 430060, China; ^2^Department of Microbiology, School of Basic Medical Science, Wuhan University, 185 Donghu Road, Wuhan, Hubei 430071, China; ^3^Institute of ENT, EENT Hospital of Fudan University, 83 Fenyang Road, Shanghai 300021, China; ^4^Laboratory of Immunology, University of Lorraine, avenue du Morvan, 54511 Vandoeuvre-lès-Nancy, France

In the article titled “Therapeutic Effectiveness and Safety of Mesotherapy in Patients with Osteoarthritis of the Knee” [1], Dr. Bo Qiu should be listed as a corresponding author along with Xianglei Wu.
